# A National Memory Clinic Survey to Assess Provision for People from Diverse Ethnic Backgrounds in England and Wales

**DOI:** 10.3390/ijerph18041456

**Published:** 2021-02-04

**Authors:** Shovanne Brown, Gill Livingston, Naaheed Mukadam

**Affiliations:** 1 Division of Psychiatry, Faculty of Brain Sciences, University College London, Gower Street, London WC1E 6BT, UK; skgtsb6@ucl.ac.uk (S.B.); g.livingston@ucl.ac.uk (G.L.); 2 Camden and Islington NHS Foundation Trust, 4 St Pancras Way, London NW1 0PE, UK

**Keywords:** dementia, ethnicity, health inequalities, memory assessment service

## Abstract

English national guidelines regarding dementia assessment and management recommend consideration of cultural and linguistic diversity when assessing people with cognitive complaints. To date there has been no assessment of adherence to these guidelines. We aimed to assess whether current services provided in memory assessment services (MAS) adhere to national policy, in their approach to the assessment and management of individuals with memory problems from minority ethnic backgrounds. We sent a survey to 213 memory services in England and Wales. Twenty MAS from seven regions responded to the survey. We found that 80% (16) provided translated resources, 70% (14) used cognitive assessment tools that are culturally sensitive and appropriate, and 65% (13) showed good use of sufficiently skilled and knowledgeable interpreters. Communication barriers, particularly language, were raised as a potential obstacle to diagnosing minority ethnic patients. Memory clinics appear to reflect national policy for the assessment and management of memory problems in minority ethnic patients. However, only a minority of services responded and they may be more engaged in considering these populations. We need wider knowledge of practice to explore how guidelines support healthcare professional’s assessment of patients from minority ethnic groups in memory service diagnostic procedures.

## 1. Introduction

The United Kingdom (UK) has a sizeable minority ethnic population making up 15% of the whole population and 39% of the London population [1]. An estimated 25,000 people in the UK living with dementia are from minority ethnic backgrounds [2]. This number is expected to double to 50,000 by 2026 [3]. With an increased prevalence of dementia [4,5,6], it is important that minority ethnic patients can access services that address their cultural and linguistic needs, facilitating timely and accurate diagnosis. In the UK, the main pathway to obtaining a dementia diagnosis for older adults is referral to a memory assessment service (MAS) where trained clinicians (usually psychiatrists) will clinically assess and diagnose dementia with the aid of imaging and neuropsychological testing where needed. All services are free at the point of care through the National Health Service (NHS).

The English National Dementia Strategy [7] consists of 17 objectives and acts as a strategic framework for health and social care professionals, to improve the quality of life for people with dementia and their carers, and anyone affected by dementia. The framework highlights the importance of providing better knowledge of dementia to reduce stigma, minimise the fear and misunderstanding of dementia, and help people understand the benefit of early diagnosis. It emphasises developing services that meet the needs of everyone, regardless of ethnic background. This is supported by supplementary policy documents such as the National Dementia Strategy Equalities Action plan [8] that states that dementia services should recognise and consider the effect that culture and social differences, such as English fluency, lack of understanding of dementia, and inaccessible and culturally insensitive services may have on ethnic communities accessing health and care services. In addition, the Prime Minister’s dementia challenge [9] was introduced in 2012 with the aim of improving health and care services, creating dementia-friendly communities and investing in dementia research.

The benefits of specific service provision for minority ethnic patients may facilitate timely diagnosis which acts as a gateway to the opportunity to access pharmacological treatments, non-pharmacological interventions for cognition and for non-cognitive symptoms and time to consider advance planning in the earlier stages of dementia. This may help to increase a patient’s quality of life allowing them to live well with dementia [7]. Despite this, an earlier study finds that some MAS provide no or limited specific memory services for patients from minority ethnic backgrounds [10]. Such studies assessing service provision for minority ethnic patients looking at only small geographical regions in England and Wales may not reflect the overall current practice of MAS, possibly due to the difference in diversity and practice within England and Wales.

Currently, we have a better understanding of the facilitators and obstacles to dementia diagnosis in minority ethnic patients. These include but are not limited to the effect that culture and ethnicity have on carer beliefs and attitudes of help-seeking, language barriers and English proficiency, the stigma of dementia diagnosis, and negative experiences and opinions of treatment within the healthcare system [11,12,13,14]. These cultural and social differences between minority ethnic groups and the majority population in help-seeking may cause delays in early diagnosis and intervention. It is therefore important to assess whether national policies are being taken up to address these obstacles and are being used to achieve good standards of care and quality of life for all patients.

We aimed to assess whether current services provided in MAS reflect national policies, regarding their approach to the assessment and management of individuals with memory problems from minority ethnic backgrounds in England and Wales.

## 2. Materials and Methods

The study did not require ethical approval as using the NHS research ethics guidelines and University College London Research Authority, the study was defined as a service evaluation (see Supplementary Materials for the exemption certificate-Figure S1). Reporting of aggregate data here is anonymous so the participants cannot be identified.

In this study, the term ethnic minority referred to any individual with a different national or cultural tradition to the majority population in the UK- White or White British. This included individuals from White other, Irish/Gypsy travellers, Indian, Pakistani, Bangladeshi, Chinese, other Asian, Mixed ethnicity, African, Afro-Caribbean and Black British backgrounds.

### 2.1. Data Collection Instrument

In the UK, strategic frameworks regarding assessment and treatment of cognitive impairment, such as the National Dementia Strategy [7], Memory Services National Accreditation Programme (MSNAP) [15], and the National Institute for Health and Care Excellence (NICE) [16] have been published with the consideration of cultural and linguistic diversity when assessing people with cognitive complaints. We searched for these and other England and Wales guidelines on dementia diagnosis and management and read each document to extract guidance specific to assessment and management of people from minority ethnic backgrounds. The main guidelines on dementia services regarding patients from minority ethnic backgrounds state that they should: provide services that meet the needs of all patients; use interpreters who are sufficiently skilled and knowledgeable, using family members of a patient only in exceptional circumstances; provide services that are accessible for all; and not rely solely on cognitive tests for diagnosis, including in circumstances where it is not possible to administer the test in a language in which the patient is sufficiently fluent. Using these published guidelines, we designed and created an online survey about memory assessment service practice.

Prior to distributing the survey, two consultant old age psychiatrists and a memory service manager evaluated it for clarity, understandability and relevance and we edited based on their feedback. Adjustments included adding a ‘not applicable’ option and free text boxes to multiple-choice questions to provide an opportunity for participants to include responses that we had not initially considered as part of the set answers. We also changed the time frame so questions asked about the last year of routine practice. This allowed participants to find data to answer the questions more easily.

The final survey consisted of 28 questions (six single selection drop-down responses, 18 multiple choice questions and four open-ended questions) and measured six main constructs: MAS patient demographics, patient referral, availability of interpreters, translated resources, cognitive assessment tools and barriers to dementia diagnosis and outreach activities (see Supplementary Materials for the survey items-Table S1). The first section of the survey concerned the patient demographic data of the MAS. The second section focused on the approximate total number of patients referred for an assessment, the percentage of patients who did not have their ethnicity recorded, the percentage of patients who were White British, and change in minority ethnic patient referral. The next section looked at the percentage of patients who did not speak English fluently and the use of family and professional interpreters. The fourth section assessed the use of cognitive assessment tools for minority ethnic patients, any limitations of use, and strategies used to support diagnosis. The final section of the survey concerned MAS outreach activities with community services, significant obstacles to diagnosing minority ethnic patients, and barriers to post-diagnostic services or interventions. The end of the survey included space for participants to provide additional comments on their experience of diagnosing and offering post-diagnostic support to minority ethnic patients in their memory clinic.

### 2.2. Survey Procedure

Members of the NHS England Dementia Clinical Network were recruited to assist in the circulation of the survey. Participants of our study were managers or clinical leads for MAS in England and Wales. They were identified through the NHS England Dementia Clinical Network, the Royal College of Psychiatrists, MSNAP and emails and telephone calls to individual services. The survey was distributed to MAS in the following regions in England and Wales: North East England, North West England, Yorkshire, West Midlands, East Midlands, South West England, South East England, East of England, Greater London, and Wales.

All MAS were sent an introductory letter by email which detailed the aim of the study, the definition of minority ethnic group, the duration of the survey, that all responses would be strictly confidential, mentioned a £50 voucher incentive, a link to the online survey and contact details of the researcher. The letter also detailed relevant instructions to aid the completion of the survey, specifically, that participants should think about the year prior to COVID-19 adjustments when routine memory assessment service activity took place. We contacted all MAS in England and Wales by email in April 2020. Data was collected between 19 April and 21 August 2020. Four weeks after sending the survey, we sent a follow-up email to all MAS, and again in mid-June for those who had not participated in the survey.

### 2.3. Data Analysis

We made demographic information non-identifiable by not reporting names of MAS and grouping the data by region of the MAS in England and Wales. To assess whether MAS were reflective of national policy regarding the assessment and management of individuals with memory problems from minority ethnic backgrounds, we grouped quantitative data into the six question constructs. We summarised the data by calculating the percentage of responses to show the proportion of MAS that provided a particular answer. Qualitative data from the open-ended questions were organised by common themes that emerged from data analysis and reported under their question subtype.

## 3. Results

### 3.1. Demographics

Of the 213 MAS contacted, 20 (9.4%) in seven out of 10 regions we sent the survey to in England and Wales responded to the survey. We received responses from MAS in the following regions: North East England (1), North West England (4), South West England (1), South East England (4), East of England (1), Greater London (8), Wales (1). In 11 (55%) of the participating MAS, ethnic minority referrals comprised less than 20% of patients seen for an initial assessment (Refer to Table 1). Almost half-8(40%) of participating MAS were based in Greater London where a greater range of diversity in patients is seen, possibly due to the higher percentage of minority ethnic groups making up the total population. The majority of MAS-13(65%) reported that in their experience, the number of minority ethnic patients attending memory services was staying the same. No MAS reported that the number of minority ethnic patients attending their memory clinic had decreased in the last year. Table 1 presents a summary of the number of responses we received from each region, the demographic makeup of the region, the percentage of ethnic minority patients that each MAS estimated they saw in the last year, the percentage of patients who did not speak English fluently, the approximate use of interpreters in one year and if the MAS provides translated resources. In general, the percentage of referrals from minority ethnic groups was similar to the expected percentage of people of minority ethnic groups in those regions, according to national surveys.

### 3.2. Availability of Interpretation in Memory Clinics

The majority of patients attending MAS were reported to speak English fluently enough to be fully assessed in English. 12 (60%) of MAS reported that less than 10% of their patients did not speak English fluently. Use of interpreters in MAS ranged from two to 300 in a year (Refer to Table 1). The percentage of referrals that required an interpreter ranged from 0.16%-15.47% of the total number of patients seen for an initial assessment. Use of interpreters was generally aligned with percentage of referrals from minority ethnic groups. In the case that a patient did not speak English fluently enough to be assessed in English, 13 (65%) of MAS used professional interpreters in more than 70% of cases when an interpreter was indicated. 13 (65%) of MAS reported using family members as interpreters in less than 10% of the cases where a patient did not speak English fluently. The most common circumstances a family member of a patient was used as an interpreter was due to a lack of availability of professional interpreters in the required language, followed by the refusal by a service user:

*“Sometimes people say they speak English but when we see them it is not sufficient to complete an assessment so we would not have booked an interpreter’’*

(MAS Greater London)

Another challenge to the provision of professional interpreters highlighted was interpreters cancelling at short notice or not attending the appointment.

*“The interpreter booked and did not arrive on the day”*

(MAS Greater London)

*“The professional interpreter cancelled in short notice”*

(MAS Greater London)

### 3.3. Provision of Translated Resources

MAS who responded offered resources in a range of languages with the most common being information about dementia diagnosis (14 responses), followed by leaflets about medication (13 responses) and information about dementia subtypes (12 responses). The three most common languages that resources were translated in were Punjabi, Urdu and Bengali. Participating MAS also offered translated materials in other languages including Russian, Farsi, Vietnamese, and Tagalog. Four MAS, (20%) did not provide any translated resources. Our findings show that 11 (55%) of MAS report that less than 20% of their referrals are patients from minority ethnic groups, with 12 (60%) reporting that less than 10% of their patients do not speak English fluently to be fully assessed in English. Of the four MAS who reported not providing translated resources, two of the services reported that approximately 0–10% of their patients attending their clinic do not speak English fluently, one reported that 21–30% of their patients do not speak English fluently, and the final MAS did not provide data on their patients English fluency (Refer to Table 1). There was a wide range in type of translated resources available and this was not related to the percentage of referrals from minority ethnic groups.

In analysing the qualitative data for these questions, a common theme that emerged was the use of the internet to access readily available translated materials if the MAS did not offer it:

*“We access all languages available on the Alzheimer’s Society website if we do not have it in house”*

(MAS Greater London)

### 3.4. Cognitive Assessment Tools

The majority–14 (70%) of respondents reported using alternative standard and validated cognitive assessment tools for minority groups with the most commonly used being the Rowland Universal Dementia Assessment Scale (RUDAS) and translated versions of the Addenbrooke’s Cognitive Examination (ACE). MAS highlighted that some limitations of using alternative cognitive assessments tools were that they had difficulty translating the assessment and interpreting alternative cognitive tests may be challenging in a high functioning patient.

*“Interpreting the results by a non-speaker of the language is difficulty. There is a lack of guides translating and explaining the cultural adaptations to the tests”*

(MAS North West England)

We found that MAS also did not solely rely on cognitive scores for diagnosis and used specific strategies to diagnose minority ethnic patients. Three key themes emerged from data analysis with regards to additional information used to support a diagnosis:

(i) Understanding of cultural beliefs
  “We always ensure we are aware of differences in cultural norms, respect for beliefs are paramount. We have at least one post that has a protected characteristic i.e., staff member must speak Punjabi fluently to enable people to attend CST in more than one language”
  (MAS Greater London)
(ii) Collateral information:
  “Seek collateral information from family, social, and health networks”
  (MAS Greater London)
(iii) Assessing patient functioning:
  “Activities of daily living assessment”
  (MAS Greater London)

### 3.5. Barriers to Dementia Diagnosis and Outreach Activities

Nine (45%) of participating MAS highlighted that the most significant obstacle to diagnosing minority ethnic patients was language barriers followed by the patient’s lack of understanding of dementia. Problems with communicating via letters written only in English were raised as potential barriers to the assessment of minority ethnic patients

*“Language has been an issue when appointment letters are sent out in English”*

(MAS North West England)

There was evidence of some engagement between MAS and community services with the most common outreach activities being with the local voluntary sector (34.3% responses), followed by community centres (21.9% responses), and partnering with places of worship (15.6%). With regards to outreach activities in MAS, 30% of respondents reported that in their experience these activities increase the number of minority ethnic patients in the service, whilst 30% reported that it has no impact. One participant stated that:

*“Raising events with specific communities does not seem to raise referral rates, but it helps with the management of those who are referred”*

(MAS Wales)

### 3.6. Additional Comments

MAS reported trying to improve their services to suit the needs of minority ethnic patients. Those based in less diverse regions seem to lack the opportunity to tailor their approaches, possibly due to low referral rates of patients from diverse backgrounds.

*“We have been focusing on trying to better address needs of our growing number of ethnic minority patients but so far with limited success- primarily due to language barriers. Our numbers are relatively small so it is proving difficult to do things other than on an individual one to one basis”*

(MAS Wales)

## 4. Discussion

This is, to our knowledge, the first national survey to evaluate if MAS adhere to national policy in their approach to the assessment and management of individuals with suspected memory problems from minority ethnic groups. Focusing on the services provided to minority ethnic patients and the experiences of diagnosis from the perspective of healthcare professionals, we found that there was a wide range in the proportion of minority ethnic patients seen in each memory service. For almost half of the memory services, most of their patients were from a White British background which is in line with what would be expected based on underlying demographics though, in some areas in London we would expect the proportion of minority ethnic patients to be much higher. This may indicate that referral rates in some regions of England and Wales may not reflect the underlying population or suggests underuse of MAS.

Our findings provide an initial indication of how well MAS reflect guidelines for dementia assessment and management and it appears that MAS are practicing in accordance to the guidelines. In general, MAS used professional interpreters, likely to be more knowledgeable and skilled in interpretation compared to family members, in the majority of cases where a patient did not speak English fluently. They considered communication barriers, particularly language, as a major obstacle to diagnosing patients from diverse backgrounds. MAS were able to recognise the obstacles faced when diagnosing minority ethnic patients which suggests a good awareness of the different cultural and linguistic needs of these patients. There was some evidence of services trying to actively overcome these obstacles, for example, the majority of participating MAS did provide resources in translated formats and also used the internet to source readily available resources if they did not have the language available. This demonstrates that MAS do attempt to reduce some of the inequalities of service provision where resources are readily available to use. However, a fifth of MAS surveyed did not provide any translated resources to their patients. It may be that MAS who reported not providing translated resources may not have the demand for these services, for example they have a low minority ethnic population in their region. If MAS do not frequently have patient referrals from minority ethnic groups, they may not have reason to provide specific services, and rather do things on a case by case basis. However, this does not mean that the MAS would be unable to provide culturally and sensitive appropriate services.

There was also some evidence of engagement between MAS and community services, although opinions were divided in whether this increased the referral rates of minority ethnic patients or if it had no impact. The majority of MAS recognised that cognitive assessment tools used for the majority population are not suitable for minority ethnic patients, due to factors such as differences in the level of education, culture, language and potentially different baseline cognitive states and so they used different culturally adapted assessment tools that were available.

Our findings reflect national policies for dementia diagnosis where MSNAP guidelines state that the use of a patient’s family member as an interpreter should only be considered in exceptional circumstances, e.g., when it is not possible to get an interpreter in time. This is because the use of a professional interpreter may provide a degree of accuracy in delivering diagnostic feedback, which may be limited by a family member’s ability to translate medical information appropriately [17]. It is encouraging to see MAS following practice guidelines and providing professional interpreters in the majority of the cases where a patient does not speak English fluently. However, a potential barrier to the assessment of minority ethnic patients in MAS raised was problems with communicating the need for interpreters. Some services said in some cases, they were unaware an interpreter would be needed until the patient arrived. In this case, they may have to re-book an appointment. This suggests that there may be a gap in communication between other parts of the dementia care pathway, patients and MAS. The addition of this topic to further surveys might establish the role of this problem in MAS to assess if it is a significant hindrance in their ability to provide professional interpretation when required. This would help to ensure patients from minority ethnic backgrounds are appropriately assessed and supports timely and accurate diagnosis. Previous research has also highlighted inequalities that exist at all stages of the diagnostic and care pathway and we need work to better connect services and ensure people from all ethnic groups have equal access and quality of assessment and care [18].

Problems with communicating via letters written only in English was raised as another potential barrier to assessment, which supports previous research that demonstrates how language proficiency may affect MAS use [11]. This is important because if patients are unaware of their referral or appointment offered, they are less likely to attend MAS, delaying diagnosis and reducing the benefit of pharmacological treatment on symptoms [13,14,19]. It is important for healthcare staff to be aware of a patient’s linguistic needs at all stages of the dementia care pathway, e.g., offer referral letters that are translated into a patient’s language, so it does not affect service provision [20]. This would help to ensure that information giving is accessible and consistent throughout the whole dementia care pathway and not just in a single service.

In line with previous research studies, it is possible that MAS in less diverse areas of England may not provide as much specific services that meet the needs of minority ethnic groups [10]. In comparison to MAS based in regions that are highly diverse, who have the opportunity to be more active in addressing key inequalities, e.g., having a protected post for staff who speak a particular language and links with local communities. This suggests that there may be a difference between areas of England and Wales in their provision of specific dementia services, however, due to our low response rate it makes it difficult to spot any trends in service provision based on regions. In regions where there is a high population of specific minority ethnic groups, it may be advisable that MAS employ staff members that have competencies in a particular language. This may be especially important where the majority of people in the region do not speak English. Long term, this may not cost more in comparison to hiring an interpreter when required. It may also ensure that some of the problems with arranging interpreters including the lack of availability of a professional interpreter in a required language, and interpreters not arriving on the day or cancelling at short notice are less common. This may provide some additional benefits which enable MAS to be more culturally sensitive on the whole. Although, it is worth noting that people from the same country may not speak the same languages and several different dialects may be spoken. Therefore, having a protected staff post for a particular language does not mean the needs of the whole group or other minority ethnic groups who are less represented in the area will be met. However, employing staff members with language competencies may break down some linguistic barriers and provide common ground for understanding.

Low referral rates of minority ethnic patients in particular regions of England and Wales may mean that there is little demand for specific dementia services such as interpreters and translated materials. With ethnic diversity becoming more evenly spread in the UK [21], MAS should have the ability and be ready to provide culturally appropriate and sensitive dementia services when needed. However, intention and ability to provide were not factors that we measured in our survey, rather we focused on current provision. The addition of this factor to a future survey could allow a better understanding of MAS adherence to national policy. For example, it would allow MAS who may not be currently providing specific services to minority ethnic patients (due to lack of demand, low referral rates, or low minority ethnic population) to provide answers about whether they have procedures, services and resources in place if requested. This would provide a more holistic understanding of whether MAS adhere to national policy in their approach to the assessment and management of individuals with memory problems from minority ethnic backgrounds.

The MSNAP [15] guidelines states that clinical staff should fulfil competencies in Higher Education Dementia Core Skills in topics including equality diversity and inclusion in dementia care. There should be an emphasis on staff working in MAS to be confident in understanding the differing needs of minority ethnic patients with dementia, e.g., through training. We were not able to assess this during the survey so this may be an important aspect to study to ensure healthcare professionals are better equipped to deal with cultural diversity.

Recent events highlighted by the Black Lives Matter movement have made societies around the world more aware of systematic racism and its drastic impact on the Black community and ethnic minorities. More than ever, it has highlighted the importance of educating and understanding the racial disparities that unfortunately still exist in our communities. In recent years, there has been increased awareness of the challenges that Black and minority ethnic communities face in dementia care. However, there is an urgent need to persist in research that addresses how we can make adaptations to interventions and services, so they are inclusive and appropriate for these communities. The impact of these events has welcomed an avenue for change in dementia research. For example, professional bodies are recognising the need to have honest and open conversations with staff working in dementia services, regarding support in addressing and accommodating the needs of minority groups [22]. Our research follows suit in that we hope to implement best practices in the future and highlight the importance of all patients having access to support and suitable services in memory clinics.

### 4.1. Limitations

We aimed to gain a broad and representative understanding of the current practice in MAS and managed to collect data from seven out of 10 regions we contacted in England and Wales. We initially intended to include Scotland in our sample but could not as we did not have a centralised list for Scottish memory services, and were unable to contact any before data collection started. The survey was sent out at the beginning of the COVID-19 pandemic and therefore it was perhaps unsurprising that response rates were low. Memory service activity was disrupted, for example, staff members may have been ill or redeployed to inpatient psychiatric and general services. We attempted to overcome this limitation by sending two follow-up emails and collating information about all memory services so we could contact them directly through telephone to increase our response rate. Understandably, this was a very difficult time and participants may not have been able to complete the survey. 40% [8] of the responding MAS were from Greater London with other regions less represented. Therefore, it may be difficult to generalise our findings to suggest that all memory clinics in England and Wales are offering services to minority ethnic patients which reflect national guidelines. Despite this, participating MAS came from areas with both high and low underlying minority ethnic populations. This may provide an indication that MAS are reflecting national guidelines and considering the cultural and linguistic needs of minority ethnic patients, regardless of region.

A potential source of bias in our study is non-response bias, where participants who were interested in the study’s research topic were more likely to participate in our survey. It could be that MAS who did not respond to the survey may have no or limited service provision for minority ethnic patients or few minority ethnic patients. Therefore, the data collected from the survey may only reflect the positive side of current practice and may lead to an over-estimation of how well MAS reflect national guidelines. We attempted to overcome this by collecting data over a number or months and by being flexible with the data collection deadline and extending it to accommodate for later responses from some of the MAS. We also ensured confidentiality by stating in our introductory letter that all responses are strictly confidential and results would be reported without naming memory services.

### 4.2. Future Research

Future research could consider further how national policy is used in MAS to support the diagnosis of minority ethnic patients. Future studies could assess MAS views on national policies such as MSNAP, the National Dementia Strategy, and NICE guidelines to see if they provide enough specific guidance for the assessment of minority ethnic patients and if they judge they cover everything necessary. For example, if the guidelines provide MAS with reasonable knowledge on how to tailor memory services with consideration of cultural and linguistic needs to meet the increasing demand for the assessment of these patients. This would provide further insight into the effectiveness of these guidelines, when used to diagnosis minority ethnic patients and provide services that support timely and accurate diagnosis and perhaps provide suggestions to use for future updates of national dementia guidelines. We could additionally survey provision for training staff in assessing diverse populations.

## 5. Conclusions

In conclusion, this study shows that in general MAS respondents recognise the linguistic and cultural needs of minority ethnic patients, attempt to overcome the recognised obstacles of diagnosing these patients and provide culturally sensitive and appropriate services including the use of professional interpreters, translated resources and use of culturally sensitive cognitive assessment tools, which reflects national policy for dementia assessment and management. Our survey suggests that there may be a disparity across England and Wales in the provision of specific dementia services tailored for minority ethnic patients that would facilitate timely and accurate diagnosis. This may be due to the population of minority ethnic groups in certain regions being low, therefore less use, or the number of patients who attend the memory clinics who do not speak English. However, this does not provide an indication on a MAS ability or readiness to provide these services when required. There is a growing understanding of the obstacles minority ethnic groups face in the dementia care pathway. It is now essential to assess the use of national policy in MAS to evaluate if strategic frameworks for dementia diagnosis and management reflect this knowledge, and provide guidelines that are specific enough to support dementia healthcare professions in the assessment of patients from minority ethnic backgrounds. This will ensure that healthcare professionals are confident in their ability to tailor services and interventions, so they are culturally appropriate and meet the needs minority ethnic patients.

## Figures and Tables

**Table 1:** Summary table with demographic information and its relation to the percentage of minority ethnic patients attending MAS and use of interpreters.

Region	Number of Responses Received in Region	% of Ethnic Minorities in Region	Number of Referrals per Year (Range)	% of Ethnic Minority Referrals	% Not Speaking English	Approximate Use of Interpreters in One Year N (% of Total Referrals)	Translated Materials
North East England	1	6.4	1000	10–19	0–10	20 (2)	2,3,4
North West England	4	12.9	420	30–39	31–40	65 (15.47)	2,3,4
			1200	10–19	0–10	2 (0.16)	2,3,4,5,6,7,8,9
			300	0–9	11–20	20 (6.6)	2,3,4,5,6,7,8,9,
			N/A	40–49	21–30	50	2,3,4,7,8
South West England	1	8.2	540	0–10	0–10	5 (0.92)	2,4
South East England	4	14.8	415	10–19	0–10	5 (1.2)	2,3,4,5,6,7,8,9
			544	10–19	0–10	10 (1.84)	0
			100	10–19	0–10	5 (5)	1,2,3,4,5,6,7,89
			800	0–10	0–10	10 (1.25)	2,3,4,6,7,8,9
East of England	1	14.6	1200	0–9	0–10	5 (0.4)	0
Greater London	8	55.1	1500	20–29	0–10	200–300(13.3–20)	2,3,4,5,7,8,9
			500	20–29	21–30	200–300(13.3–20)	2,3,4,5,6,7,8,9
			730	60-69	21-30	N/A	10
			700	50-59	0-10	60 (8.6)	2,3,4,7,8,9
			946	60-69	0-10	N/A	0
			900	30-39	21-30	200 (22.2)	0
			430	70-79	0-10	20 (4.7)	10
			1600	10-19	11-20	200 (12.5)	2
Wales	1	6.8	1540	0–9	10–19	96 (6.2)	2,3,4,5,6,7,8,9
**Total**	20						

Note: N/A- MAS who report not storing information to provide data to answer the question. Provision of translated materials options: 0-No translated resources, 1-Online videos, 2-Information about dementia diagnosis, 3-Information about dementia subtypes, 4-Leaflets about medication, 5-Leaflets about other psychosocial treatments including Cognitive Stimulation Therapy CST, 6-Information about participation in research, 7-Information about lasting power of attorney, 8-Information about advanced care planning, 9-Leaflets about driving and dementia, 10-Other.

## Data Availability

Data are available from the authors upon request.

## Supplementary Materials

The following are available online at https://www.mdpi.com/1660-4601/18/4/1456/s1.
