# Supplementary material for: A National Memory Clinic Survey to Assess Provision for People from Diverse Ethnic Backgrounds in England and Wales
Source: Int J Environ Res Public Health. 2021 Feb 4;18(4):1456. doi: 10.3390/ijerph18041456 (PMC7913949; doi:10.3390/ijerph18041456)
Supplement: Supplementary file 1 [file ijerph-18-01456-s001.pdf]

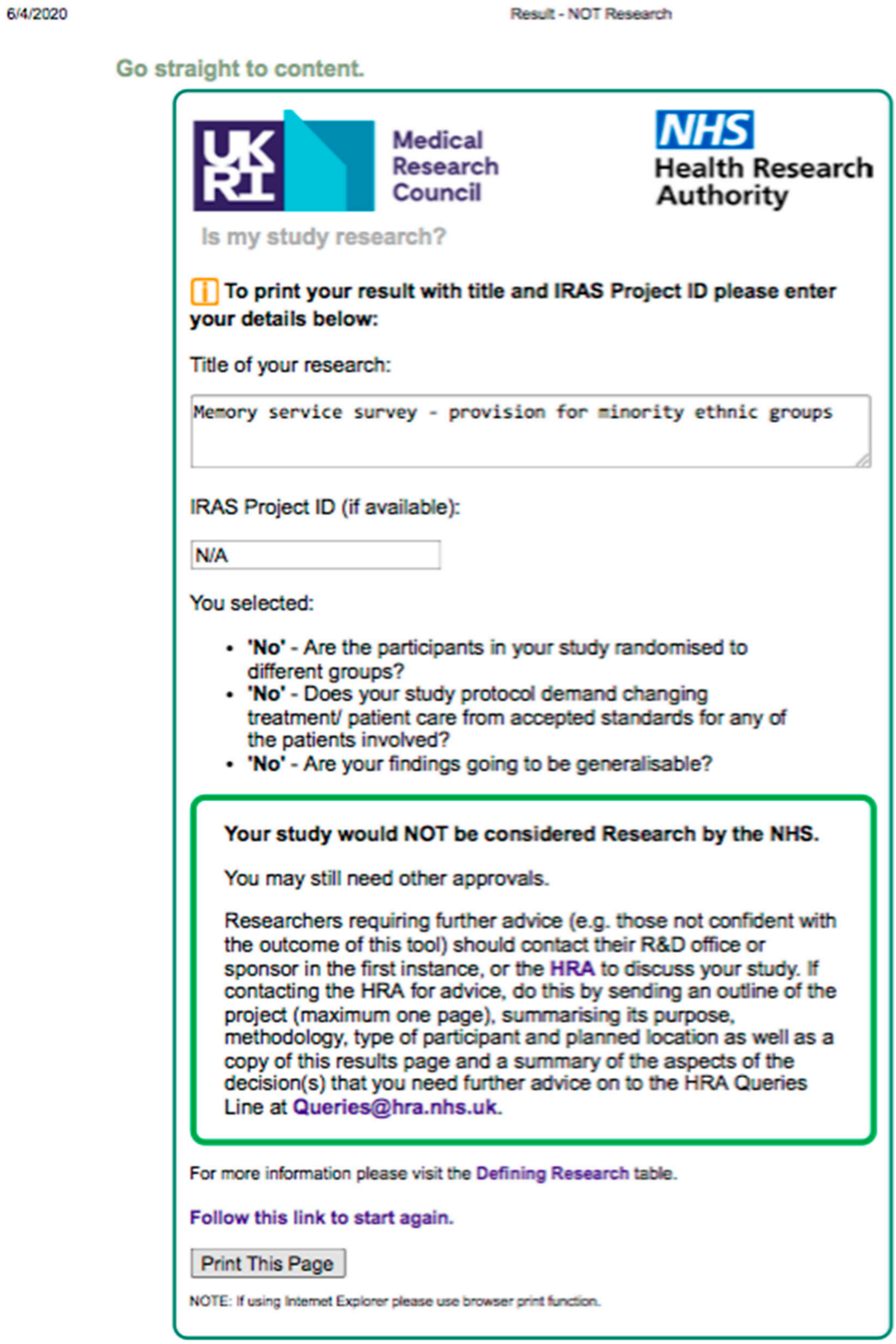

Go straight to content.

UKRI Medical Research Council

NHS Health Research Authority

Is my study research?

To print your result with title and IRAS Project ID please enter your details below:

Title of your research:

Memory service survey - provision for minority ethnic groups

IRAS Project ID (if available):

N/A

You selected:

- **'No'** - Are the participants in your study randomised to different groups?
- **'No'** - Does your study protocol demand changing treatment/ patient care from accepted standards for any of the patients involved?
- **'No'** - Are your findings going to be generalisable?

Your study would NOT be considered Research by the NHS.

You may still need other approvals.

Researchers requiring further advice (e.g. those not confident with the outcome of this tool) should contact their R&D office or sponsor in the first instance, or the **HRA** to discuss your study. If contacting the HRA for advice, do this by sending an outline of the project (maximum one page), summarising its purpose, methodology, type of participant and planned location as well as a copy of this results page and a summary of the aspects of the decision(s) that you need further advice on to the HRA Queries Line at **Queries@hra.nhs.uk**.

For more information please visit the **Defining Research** table.

Follow this link to start again.

Print This Page

NOTE: If using Internet Explorer please use browser print function.

Figure S1. Ethical approval exemption certificate

Table S1. Survey items.

NHS Memory Service Survey

Please note that the term ‘ethnic minority’ in this survey refers to any individual who has different national or cultural traditions to the main population in the UK- White or White British. This includes: White other, Irish/Gypsy travelers, Indian, Pakistani, Bangladeshi, Chinese, Other Asian, Mixed ethnicity, African, Afro-Caribbean or Black British

1. **Please state the name of your memory service.**

2. **Where in the UK is your memory clinic based?**

Scotland
Northern Ireland
Wales
North East England
North West England
Yorkshire
West Midlands
East Midlands
South West England
South East England
East of England
Greater London

3. **In the last year of routine practice (before Coronavirus adjustments), approximately how many patients in total were seen for an initial assessment?**

4. **Please make an informed estimate of the percentage of patients who did not have their ethnicity recorded in the last year**

0-10%
11-20%
21-30%
31- 40%

41- 50%
51- 60%
61- 70%
71- 80%
81- 90%
91- 100%

5. **Out of the total number of patients who were seen for an initial assessment, approximately what percentage were white British?**

0-10%
11-20%
21-30%
31-40%
41-50%
51-60%
61-70%
71-80%
81-90%
91-100%

6. **In your experience, would you say that there has been a change in the number of ethnic minority patients attending your memory clinic in the last one year?**

Yes- increasing

Staying the same

Yes- decreasing

7. **From your experience, approximately what percentage of patients who attend your memory clinic do not speak English fluently enough to be fully assessed in English?**

0-10%
11-20%
21-30%
31-40%
41-50%
51-60%
61-70%
71-80%
81-90%
91-100%

8. **In the last one year, approximately how many times was an interpreter used in your memory clinic i.e., at people's homes or in the clinic?**

9. **The Memory Service Accreditation Programme states that resources should be available to support the assessment and diagnosis of patients. This includes the use of interpreters. Of the number of patients who attend the memory clinic and do not speak English fluently, approximately what percentage use:**

   a) a family interpreter at appointments?
   0-10%
   11-20%
   21-30%
   31-40%
   41-50%
   51-60%
   61-70%
   71-80%
   81-90%
   91-100%

10. **Of the number of patients who attend the memory clinic and do not speak English fluently, approximately what percentage use:**

    b) a professional interpreter at appointments?
    0-10%
    11-20%
    21-30%
    31-40%
    41-50%
    51-60%
    61-70%
    71-80%
    81-90%
    91-100%

11. **Please tick the circumstances under which a family member was used as an interpreter in your memory clinic (Tick ALL that apply)**

12. **Of the reasons ticked above, which are the two most frequent reasons?**

The service user turned down the use of a professional interpreter

The memory clinic did not deem it necessary to use a professional interpreter

Financial constraint

There was no availability of interpreters in the required language

A professional interpreter was not available in the required timeframe

Other reason (please specify)

13. **Does your memory clinic provide information to patients? If so, please tick the resources that your memory clinic offers to patients (Tick ALL that apply)**

Online videos

Information about dementia diagnosis

Information about dementia subtypes

Leaflets about medication

Leaflets about other psychosocial treatments including CST

Information about participation in research

Information about lasting power of attorney

Information about advanced care planning

Leaflets about driving and dementia

Other (please specify)

14. **Does your memory clinic provide information in a translated format? If so, please tick the resources that your memory clinic offers translated versions of (Tick ALL that apply)**

Online videos

Information about dementia diagnosis

Information about dementia subtypes

Leaflets about medication

Leaflets about other psychosocial treatments including CST

Information about participation in research

Information about lasting power of attorney

Information about advanced care planning

Leaflets about driving and dementia

Other (please specify below)

15. **What languages does your memory clinic offer translated materials in? (Tick ALL that apply)**

Cantonese

Polish

Punjabi

Urdu

Bengali

Gujarati

Arabic

French

Portuguese

Spanish

Turkish

Mandarin

German

Greek

Somali

Yoruba

Other (please specify)

16. **What is the most common cognitive assessment used for your clinic's majority population (i.e., white British) patients (Tick ONE)?**

Mini Mental State exam

Addenbrooke's Cognitive test

The Montreal Cognitive Assessment

General Practitioner Assessment of Cognition

The Rowland Universal Dementia Assessment scale

Other (please specify)

17. **Some patients from ethnic minority backgrounds may be disadvantaged being assessed using some cognitive tests. Does your memory service use other**

standard and validated cognitive tests for patients from ethnic minority backgrounds?

Yes (please specify which tests)

No

Cognitive tests

18. **If 'yes' to question 17, are there any limitations of using the alternative cognitive assessment specified?**

Not applicable

Limitations of alternative cognitive assessments

19. **Does your memory clinic use any strategies when trying to diagnose ethnic minority patients with dementia?**

Yes

No

Please provide more information on the strategies used

20. **Several obstacles are faced when diagnosing patients from ethnic minority patients in memory clinics.**
    In your experience, which obstacles are most significant when diagnosing a patient from an ethnic minority background? (Tick ALL that apply)

Language barriers

Cultural differences

Stigma of diagnosis

Lack of understanding of dementia

Please feel free to expand or discuss other obstacles that have not been mentioned above

21. **To engage and raise awareness of memory disorders in ethnic minority groups, how does your memory clinic network with community services? (Tick ALL that apply)**

Visits to community centres

Drop in sessions at memory service

Partnering with places of worship

Local voluntary sector

Not applicable

Other (please specify)

Other

22. **In your experience, what impact does networking with community services have on engaging and raising awareness in ethnic minority patients attending your memory clinic?**

Increases the number of ethnic minority patients attending

Has no impact

Decreases the number of ethnic minority patients attending

Not applicable

23. **Do you feel that the 'do not attend' rates of ethnic minority patients are different to the majority population?**

Less than

Equal to

More than

Do not know

24. **In your experience, what are the most common reasons that ethnic minority patients do not attend appointments?**

25. **Which services are most difficult for ethnic minority patients to access due to barriers e.g., language? (Tick ALL that apply)**

CST

Carers Navigation

Carers Psychoeducation

Other (please specify)

Other service

26. **If the services are offered in an alternative form, please explain how services or interventions are adapted in your memory clinic to make them more accessible to ethnic minority patients**

Not applicable

27. **Does your memory clinic ask ethnic minority patients to be involved in research post diagnosis?**

Yes

No

28. **What post diagnostic services does your memory clinic offer to ethnic minority patients? (Tick ALL that apply)**

Local support services

Connect with dementia advisors

Advanced care planning

Referral to social services if appropriate

Other (please specify)

Other post-diagnostic service

29. **If you have any additional comments you would like to make regarding your experience of diagnosing ethnic minority patients or offering post diagnostic interventions in memory clinics please type them below**

Thank you for your time and for completing our survey.
